# Supplementary material for: Charge self-regulation in 1T'''-MoS2 structure with rich S vacancies for enhanced hydrogen evolution activity
Source: Nat Commun. 2022 Oct 10;13:5954. doi: 10.1038/s41467-022-33636-8 (PMC9550810; doi:10.1038/s41467-022-33636-8)
Supplement: Supplementary file 1 — Supplementary Information [file 41467_2022_33636_MOESM1_ESM.pdf]

Supplementary Information

Charge self-regulation in 1T'''-MoS_2 structure with rich S vacancies for enhanced hydrogen evolution activity

Xiaowei Guo, [1,2#] *Erhong Song,*[1,2#] *Wei Zhao,*[1,2#] *Shumao Xu,*[1] *Wenli Zhao,*[3] *Yongjiu Lei,*[4] *Yuqiang Fang,*[1,2] *Jianjun Liu*[1,2,5]* *and Fuqiang Huang*[1,2,6]*

[1]State Key Laboratory of High Performance Ceramics and Superfine Microstructure, Shanghai Institute of Ceramics, Chinese Academy of Sciences, Shanghai 200050, P. R. China

[2]Center of Materials Science and Optoelectronics Engineering, University of Chinese Academy of Science, Beijing 100049, China

[3]School of Physical and Mathematical Sciences, Nanjing Tech University, Nanjing 211800, China

[4]Materials Science and Engineering, King Abdullah University of Science and Technology (KAUST), Thuwal 23955-6900, Saudi Arabia

[5]Shanghai Institute of Materials Genome, 99 Shangda road, Shanghai 200444, P. R. China

[6]State Key Laboratory of Rare Earth Materials Chemistry and Applications, College of Chemistry and Molecular Engineering, Peking University, Beijing 100871, P.R. China

#These authors contributed equally to this work.

*Correspondence and requests for materials should be addressed to J. L. (email: jliu@mail.sic.ac.cn) and F. H. (email: huangfq@pku.edu.cn)

Supplementary figures

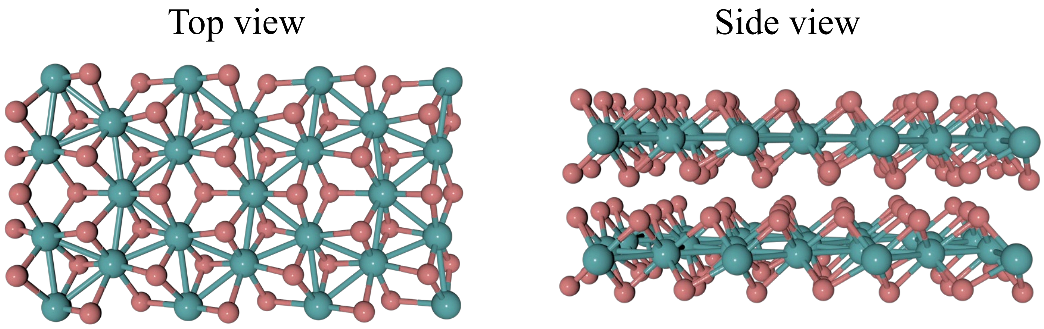

Supplementary Figure 1. Atomic structure diagram of 1T'''-MoS_2. The top view and side view of 1T'''-MoS_2.

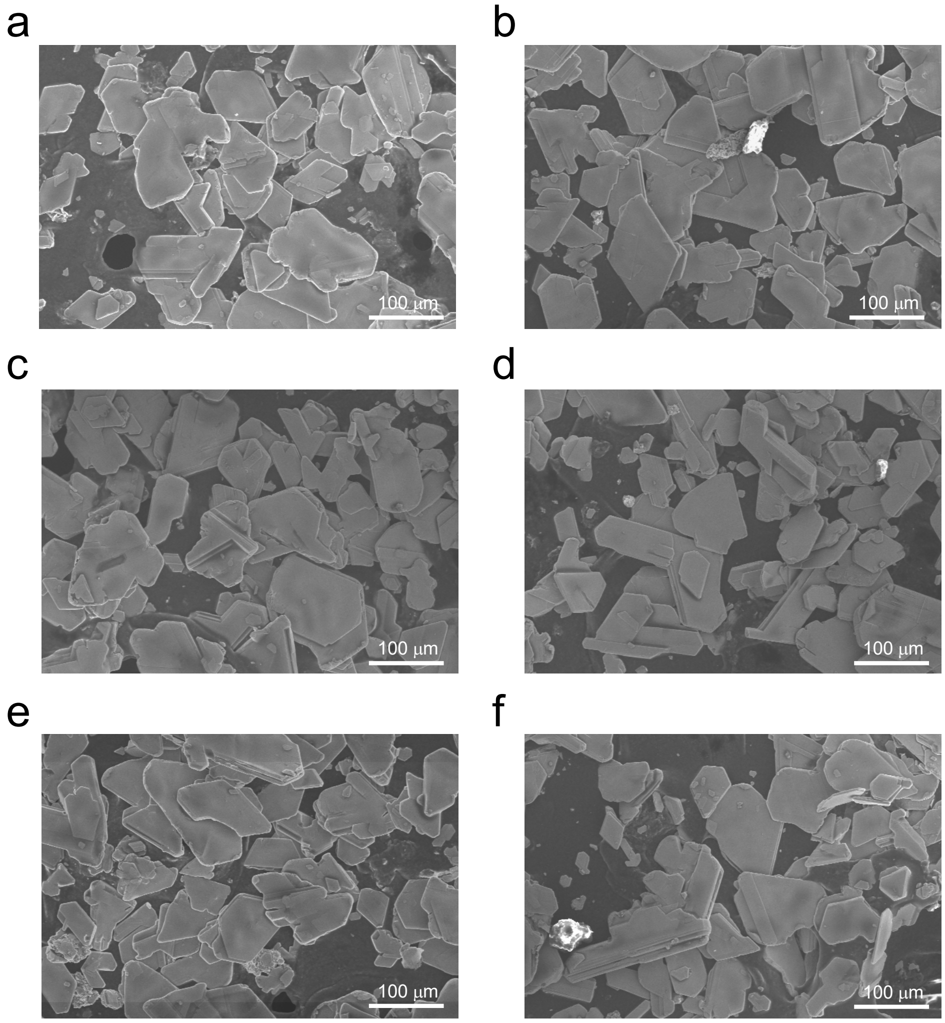

Supplementary Figure 2. SEM images of MoS_2. (a-f) The layered structures correspond to reference 2H-MoS_2-V_S and 1T'''-MoS_2-V_S (V_S = 2.0%, 7.7%, 10.6%, 17.9%, 22.9%).

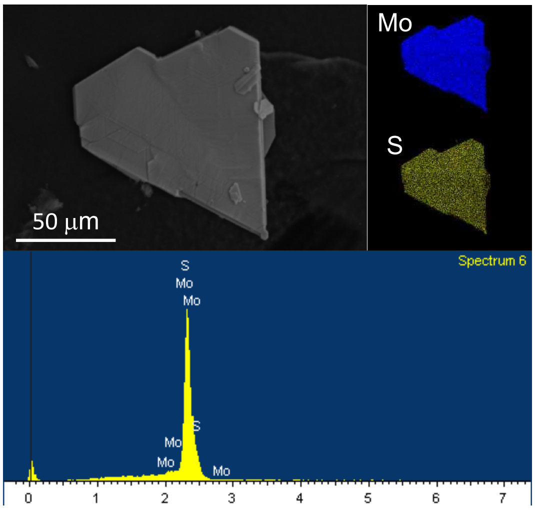

Supplementary Figure 3. SEM imagine, mapping and EDS results. 1T'''-MoS_2-10.6% in this test is consistent with the sample in Supplementary Table 5.

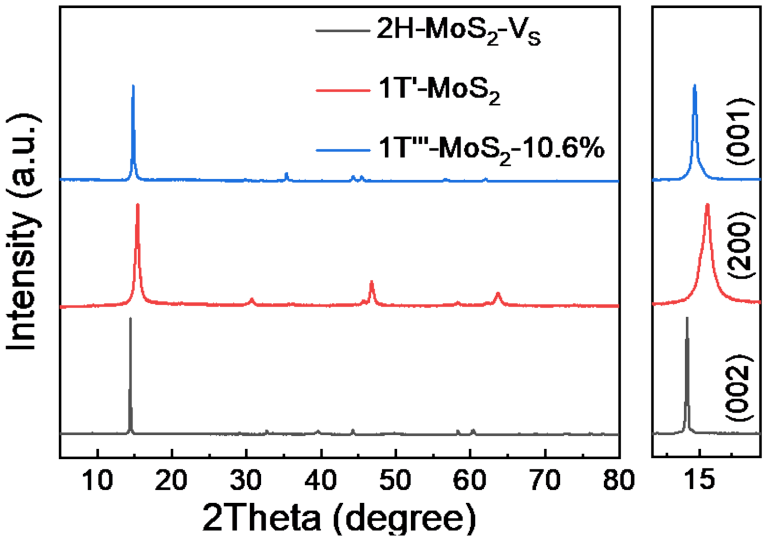

Supplementary Figure 4. XRD patterns of MoS_2. XRD patterns of the reference 2H-MoS_2-V_S, 1T'-MoS_2 and 1T'''-MoS_2-10.6%.

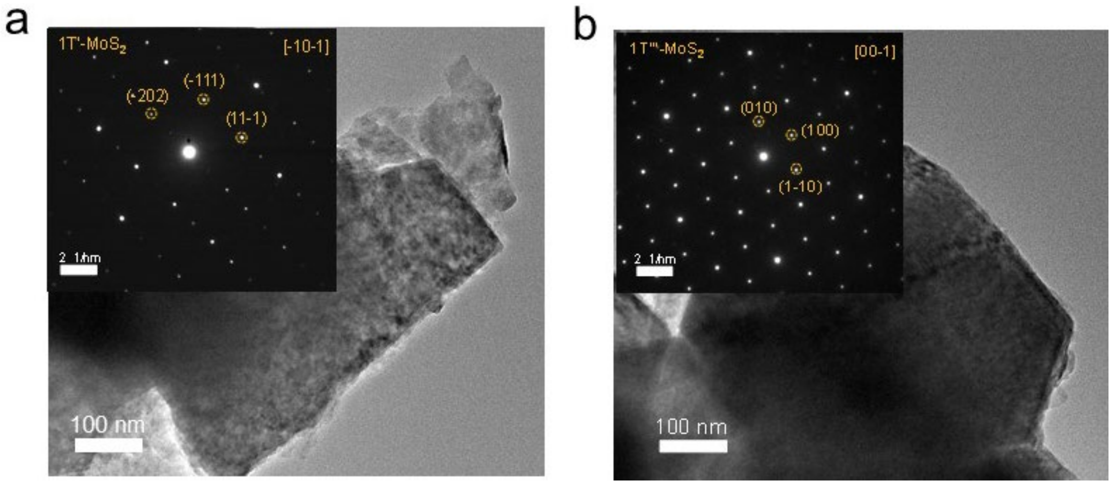

Supplementary Figure 5. HRTEM images of MoS_2. (a) 1T'-MoS_2 and (b) 1T'''-MoS_2-10.6% sheet and the corresponding SAED patterns.

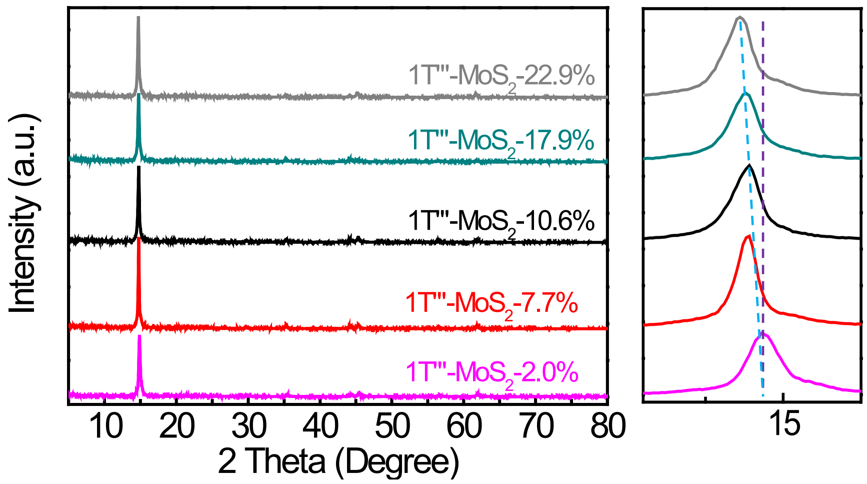

Supplementary Figure 6. XRD patterns of different 1T'''-MoS_2-V_S samples. Sulfur vacancy concentration is 2.0%, 7.7%, 10.6%, 17.9% and 22.9%, respectively.

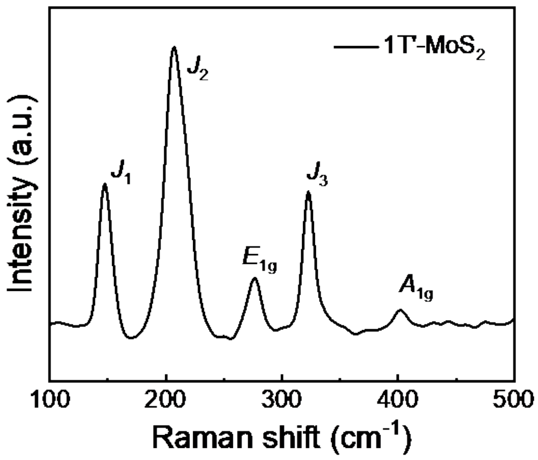

Supplementary Figure 7. Characterization of Raman spectrum. These characteristic peaks are consistent with the superlattice structure of the 1T'-MoS_2 sheet.

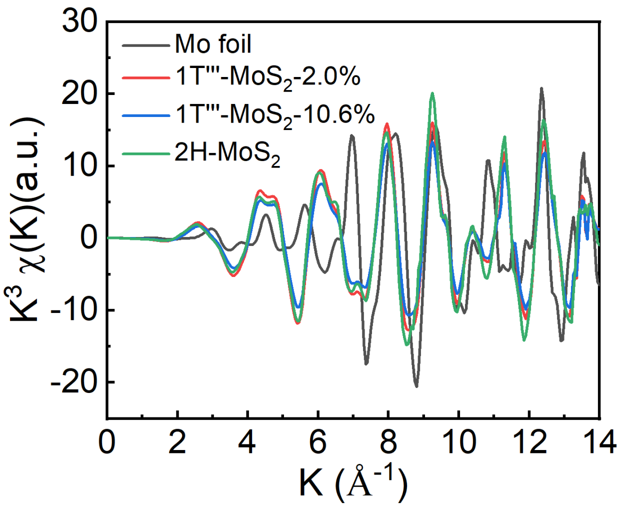

Supplementary Figure 8. k^3-weighted EXAFS oscillations of MoS_2. These samples correspond to Mo foil, 2H-MoS_2, 1T'''-MoS_2-2.0% and 1T'''-MoS_2-10.6%, respectively.

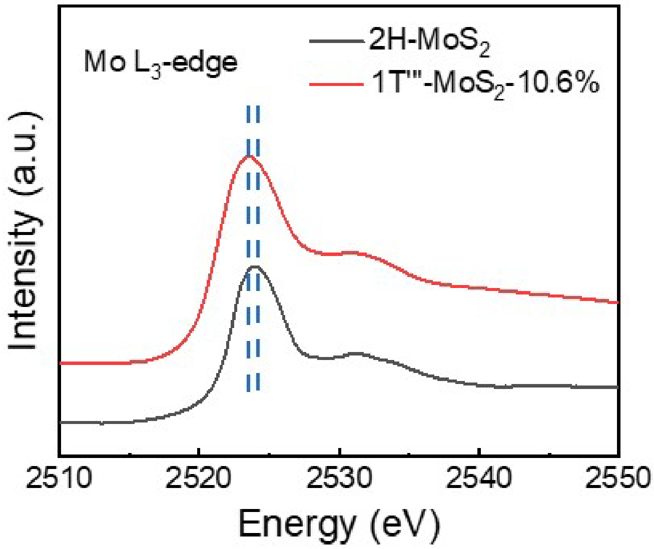

Supplementary Figure 9. Mo L_3-edges XANES spectra of MoS_2. These samples correspond to 2H-MoS_2 and 1T'''-MoS_2-10.6%, respectively.

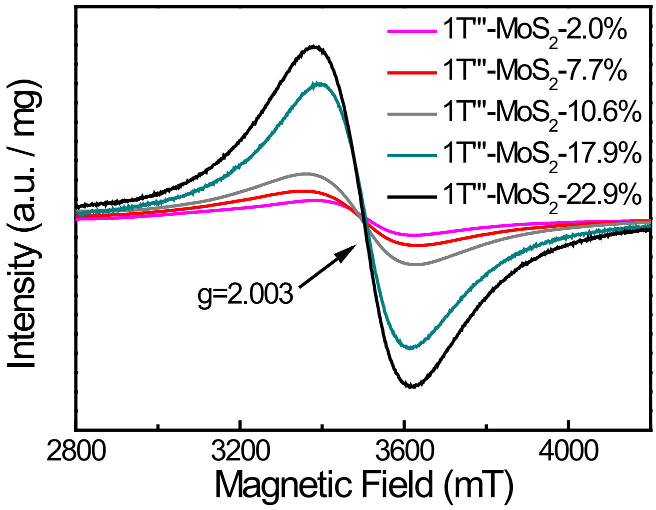

Supplementary Figure 10. Room-temperature electron paramagnetic resonance (EPR) spectra of different 1T'''-MoS_2-V_S samples. The different sulfur vacancy concentrations are 2.0%, 7.7%, 10.6%, 17.9%, 22.9%, respectively.

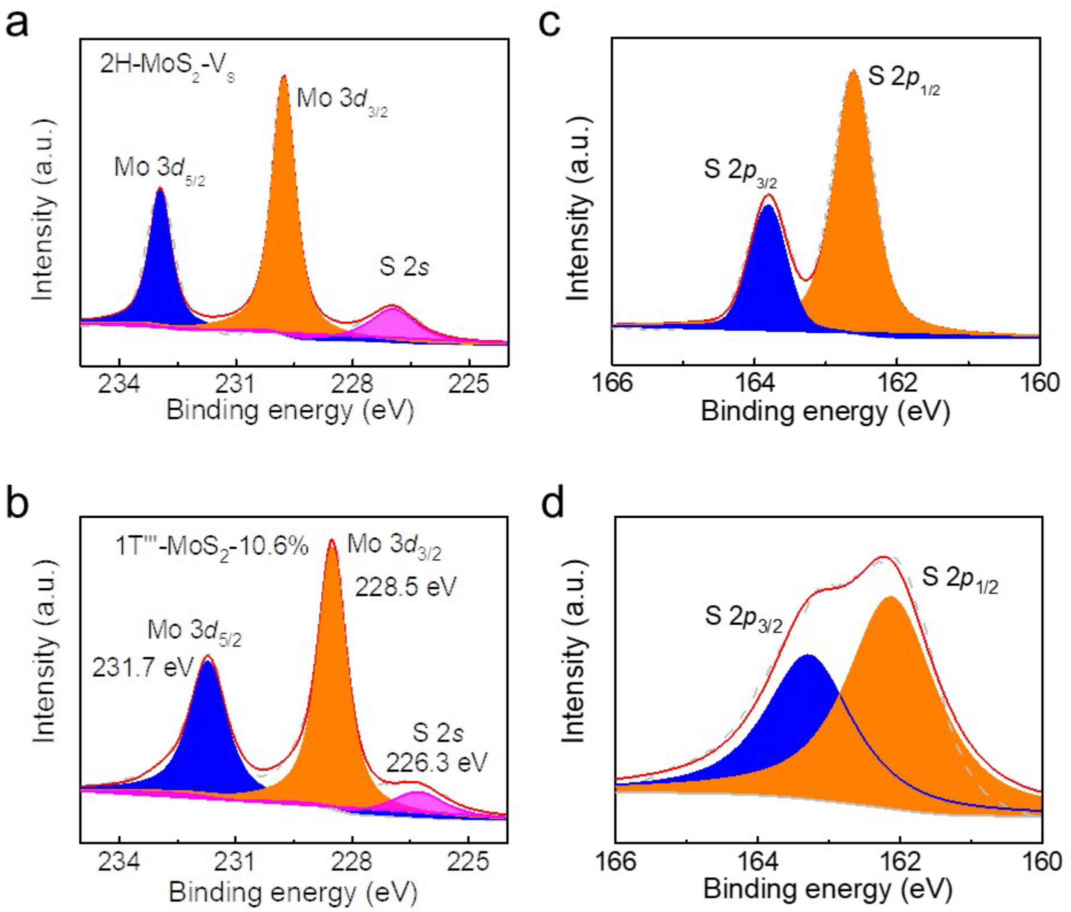

Supplementary Figure 11. Mo 3*d* region and S 2*s* region of XPS spectra. The fitted curves of (a) Mo 3*d* of 2H-MoS_2-V_S, (b) Mo 3*d* of 1T'''-MoS_2-10.6%, (c) S 2*p* of 2H-MoS_2-V_S and (d) S 2*p* of 1T'''-MoS_2-10.6%.

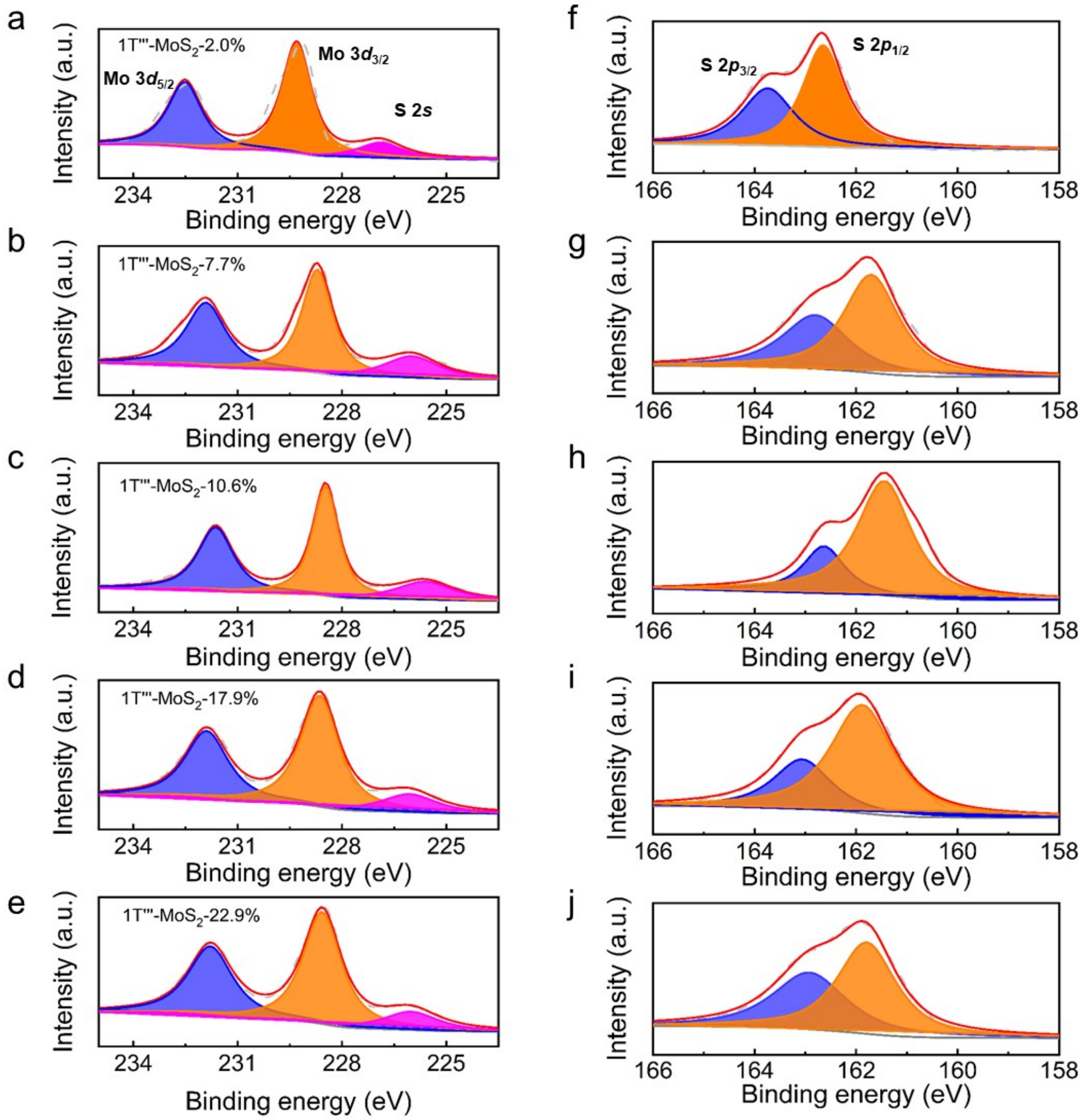

Supplementary Figure 12. The Mo 3*d* and S 2*p* of XPS spectra. The fitted curves of Mo 3*d* of (a) 1T'''-MoS_2-2.0%, (b) 1T'''-MoS_2-7.7%, (c) 1T'''-MoS_2-10.6%, (d) 1T'''-MoS_2-17.9%, and (e) 1T'''-MoS_2-22.9%. The fitted curves of S 2*p* of (f) 1T'''-MoS_2-2.0%, (g) 1T'''-MoS_2-7.7%, (h) 1T'''-MoS_2-10.6%, (i) 1T'''-MoS_2-17.9%, and (j) 1T'''-MoS_2-22.9%.

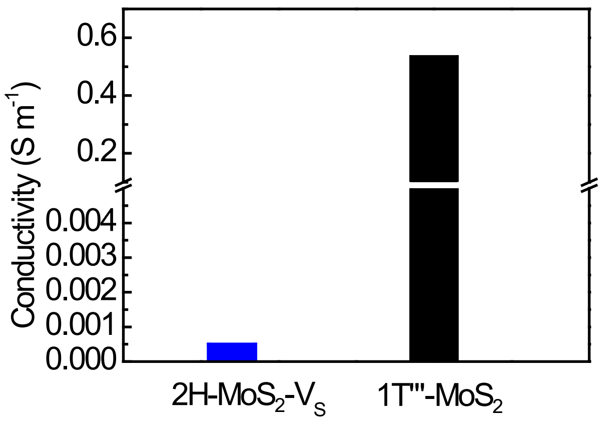

Supplementary Figure 13. The conductivity of bar chart at room temperature. Comparison between the reference 2H-MoS_2-V_S and 1T'''-MoS_2-10.6%.

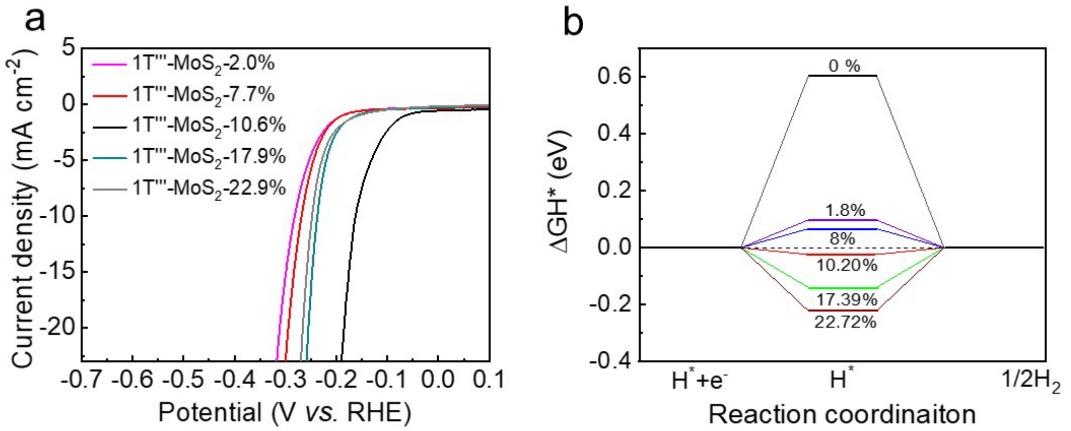

Supplementary Figure 14. Correlation of HER activity of 1T'''-MoS_2-V_S with different S vacancy concentrations between experiment and calculation. (a) Linear sweep polarization curves of 1T'''-MoS_2-V_S (V_S = 2.0%, 7.7%, 10.6%, 17.9%, 22.9%). (b) ΔG_{H*} *vs.* the reaction coordination of HER for the S vacancies range of 0-22.72%.

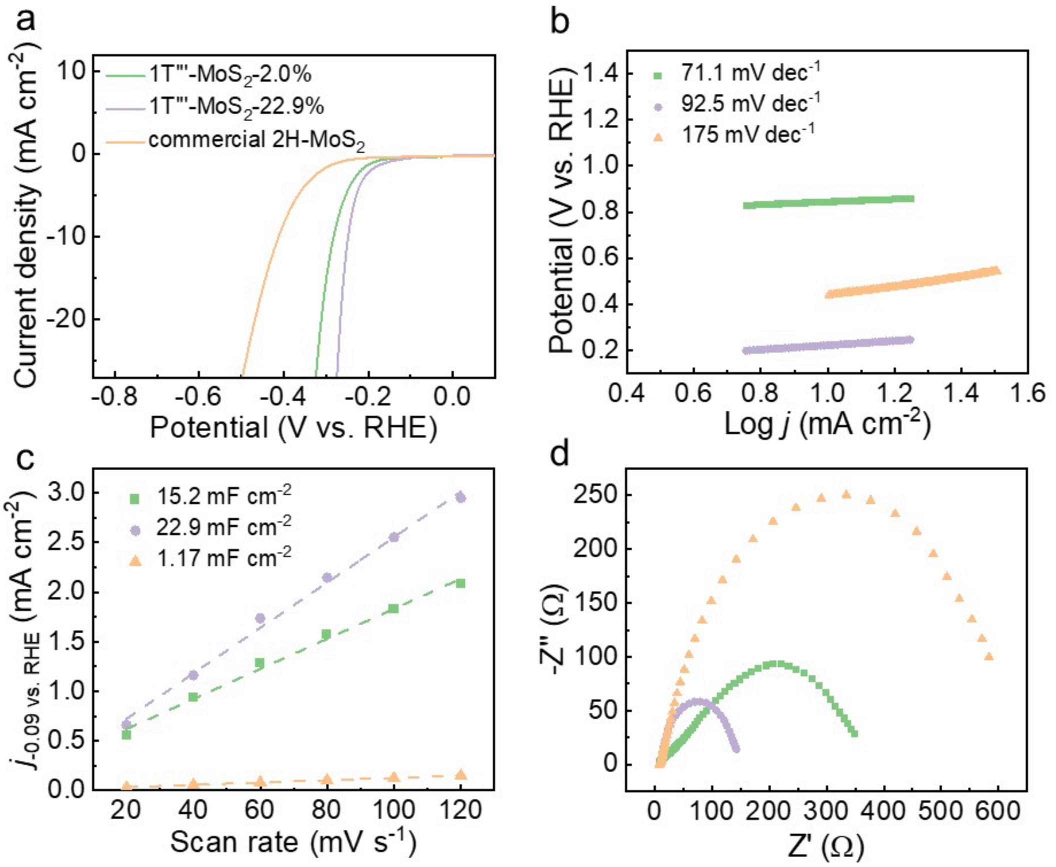

Supplementary Figure 15. HER performance in 0.5 M H_2SO_4. (a) Linear sweep polarization curves of 1T'''-MoS_2-V_S (V_S = 2.0%, 22.9%) and commercial 2H-MoS_2. (b) The corresponding Tafel curves from the polarization curves. (c) Plots of current density difference against scan rates, *j* is the difference between anodic and cathodic current densities at -0.09 V (*vs. RHE*). In the plot, the capacitance was normalized by the geometric surface area of electrodes. (d) Nyquist plots of 1T'''-MoS_2-V_S (V_S = 2.0%, 22.9%) and commercial 2H-MoS_2.

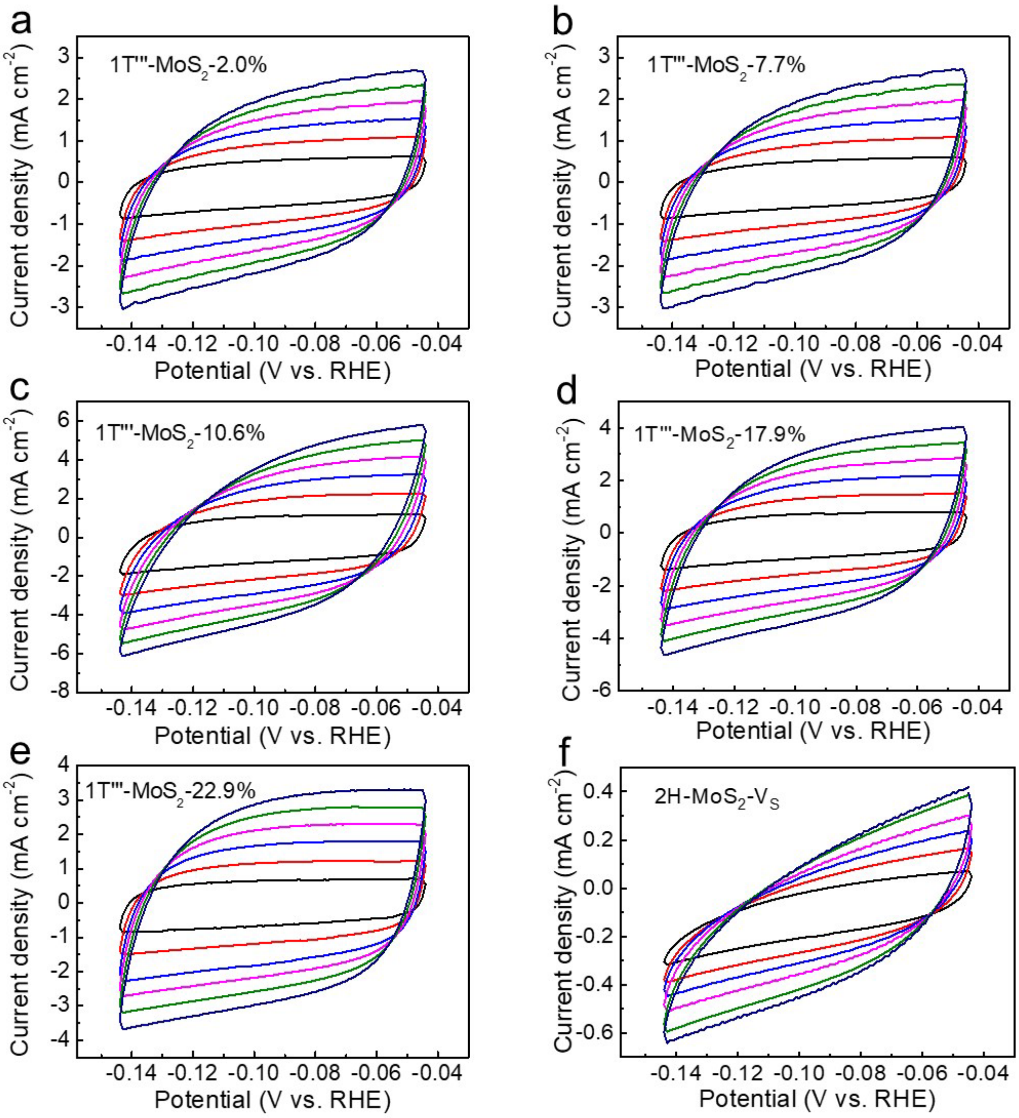

Supplementary Figure 16. The cyclic voltammograms (CVs) during electrochemical test. The as-prepared samples correspond to (a) 1T'''-MoS_2-2.0%, (b) 1T'''-MoS_2-7.7%, (c) 1T'''-MoS_2-10.6%, (d) 1T'''-MoS_2-17.9%, (e) 1T'''-MoS_2-22.9%, (f) 2H-MoS_2-V_s.

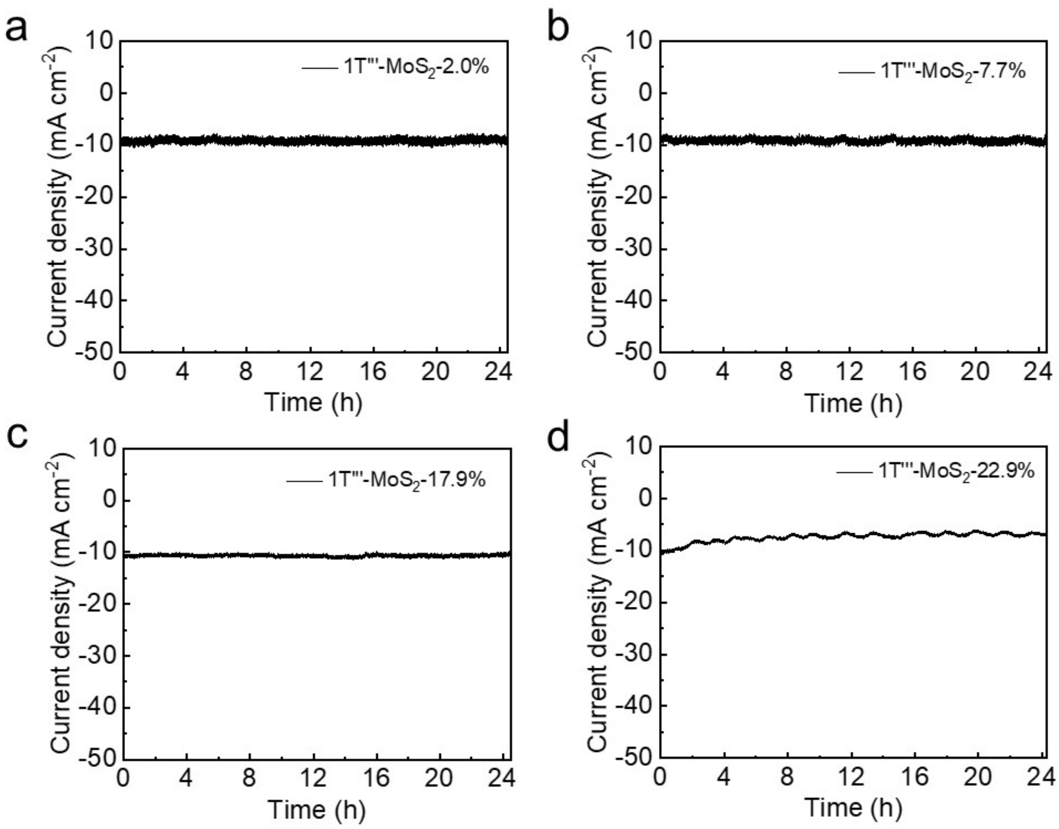

Supplementary Figure 17. The long-term durability tests of 1T'''-MoS_2 with different S vacancy concentrations. The S vacancy concentrations correspond to (a)2.0%, (b)7.7%, (c)17.9% and (d)22.9%, respectively.

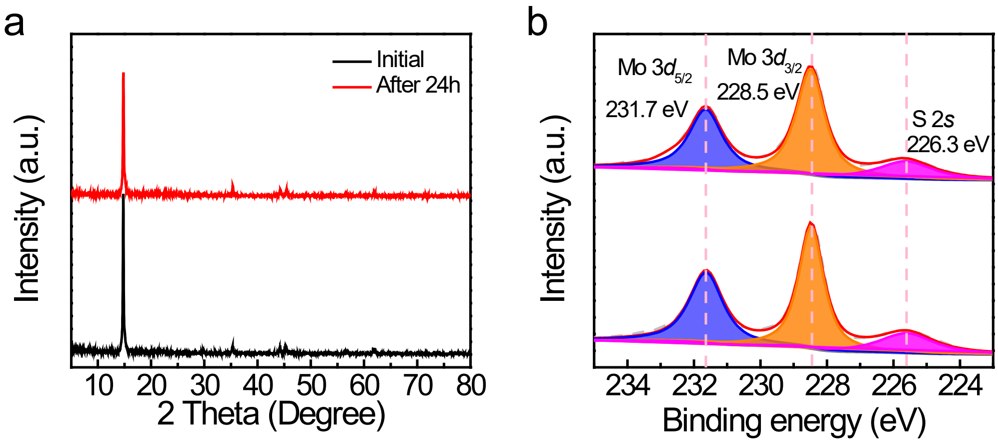

Supplementary Figure 18. The XRD and Mo 3*d* of XPS spectra before and after the electrochemical test. (a) XRD patterns and (b) the fitted curves of Mo 3*d* of 1T'''-MoS_2-10.6%.

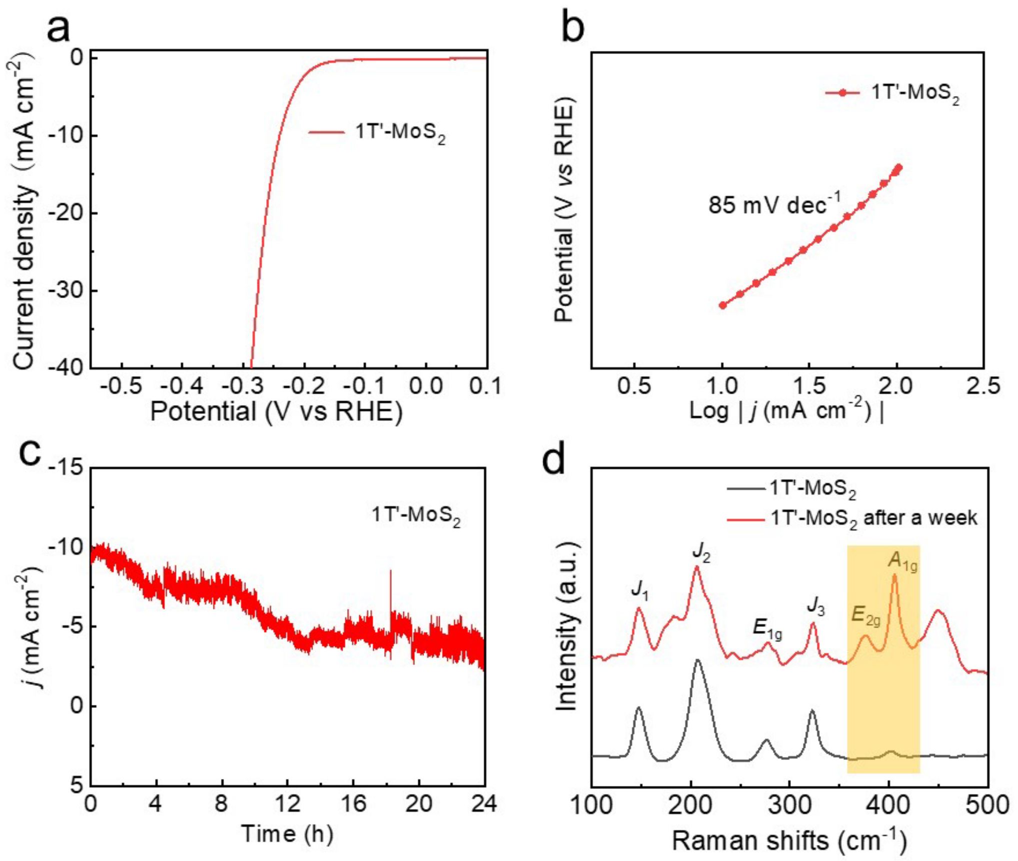

Supplementary Figure 19. HER performance measurements and Raman spectra before and after one week of storage at room temperature. (a) linear sweep polarization curve, (b) Tafel curve and (c) the long-term durability test. (d) Raman spectra of 1T'-MoS_2 before and after one week.

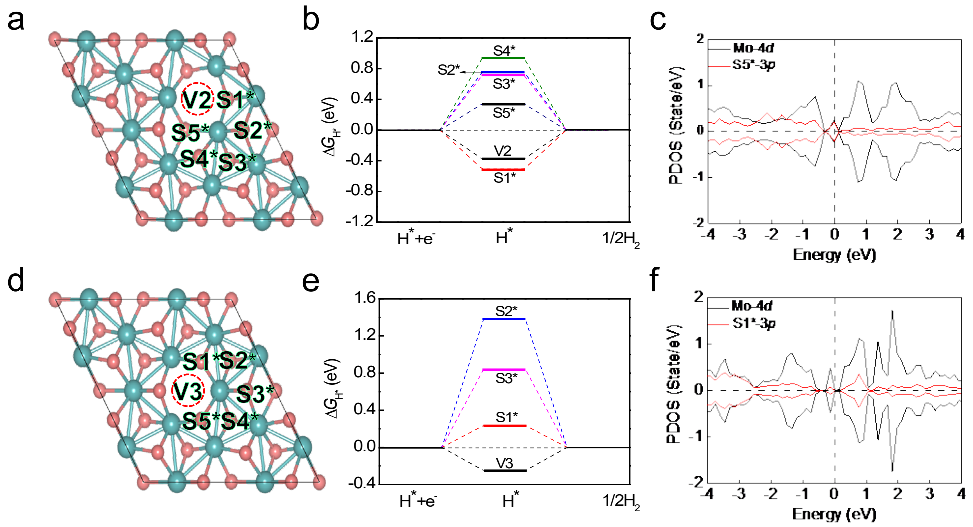

Supplementary Figure 20. Calculated hydrogen adsorption free energies and electronic structure of multiple active sites in 1T'''-MoS_2-V2, 1T'''-MoS_2-V3. (a, d) The optimized structure and of 1T'''-MoS_2-V2, 1T'''-MoS_2-V3. (b, e) ΔG_{H*} in different exposed S atoms around Mo in 1T'''-MoS_2-V2, 1T'''-MoS_2-V3. (c, f) Projected density of state (PDOS) before H absorbed on S5 of 1T'''-MoS_2-V2 and S1 of 1T'''-MoS_2-V3.

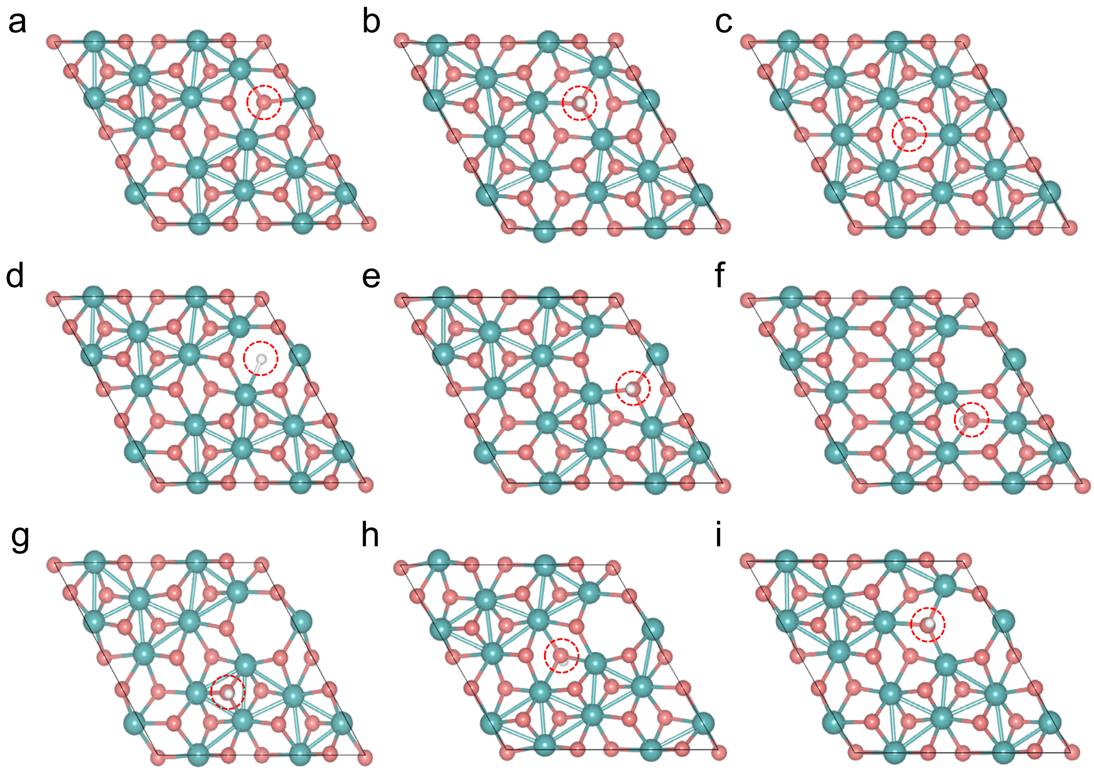

Supplementary Figure 21. Optimized structure of H absorbed on pristine 1T'''-MoS_2 and 1T'''-MoS_2-V1. (a-c) Optimized structure of H absorbed on S1, S2, S3 atoms of pristine 1T'''-MoS_2; (d-i) Optimized structure of H absorbed on V1, S2, S3, S4, S5 and S6 atoms of 1T'''-MoS_2-V1.

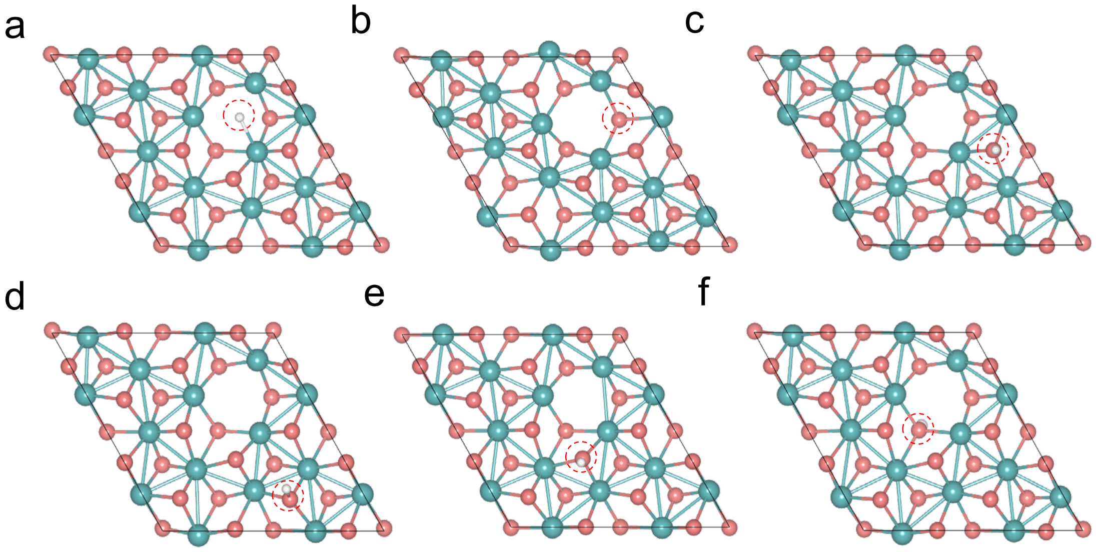

Supplementary Figure 22. Optimized structure of H absorbed on 1T''-MoS_2-V2. (a-f) H absorbed on V2, S1, S2, S3, S4, S5 atoms of 1T'''-MoS_2-V2.

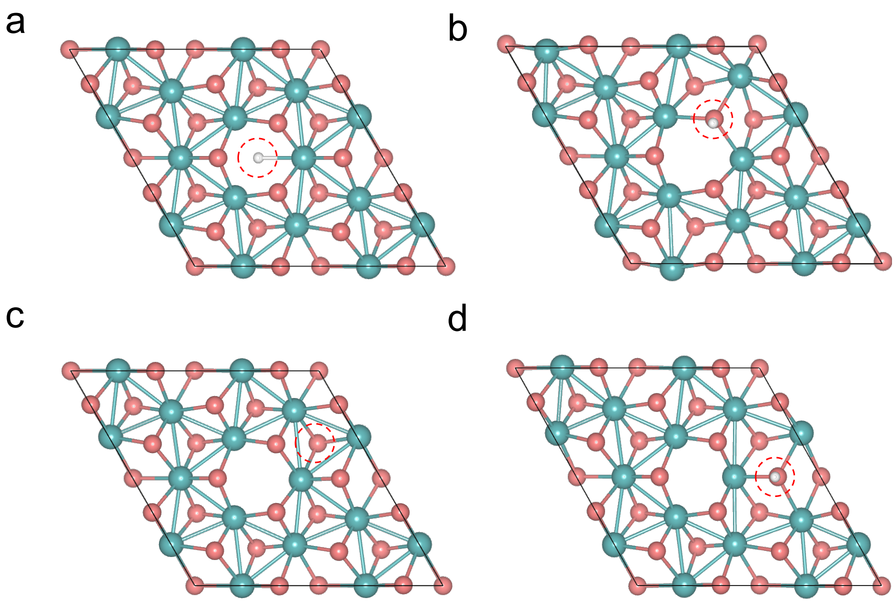

Supplementary Figure 23. Optimized structure of H absorbed on 1T'''-MoS_2-V3. (a-d) H absorbed on V3, S1, S2, S3 atoms of 1T'''-MoS_2-V3.

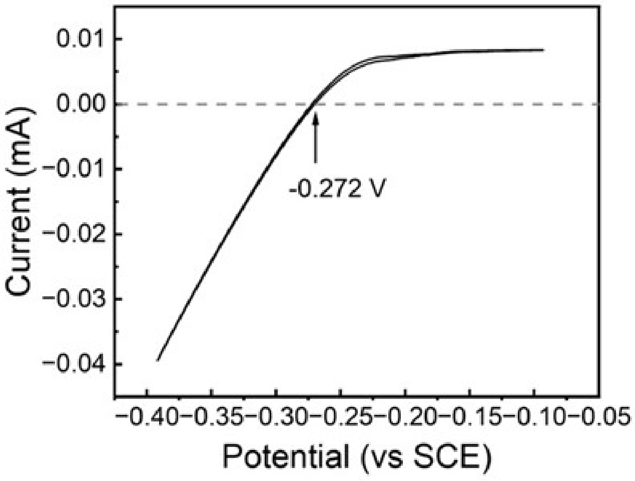

Supplementary Figure 24. The calibration curve of SCE reference electrode. In 0.5 M H_2SO_4, the zero current point is at -0.272 V.

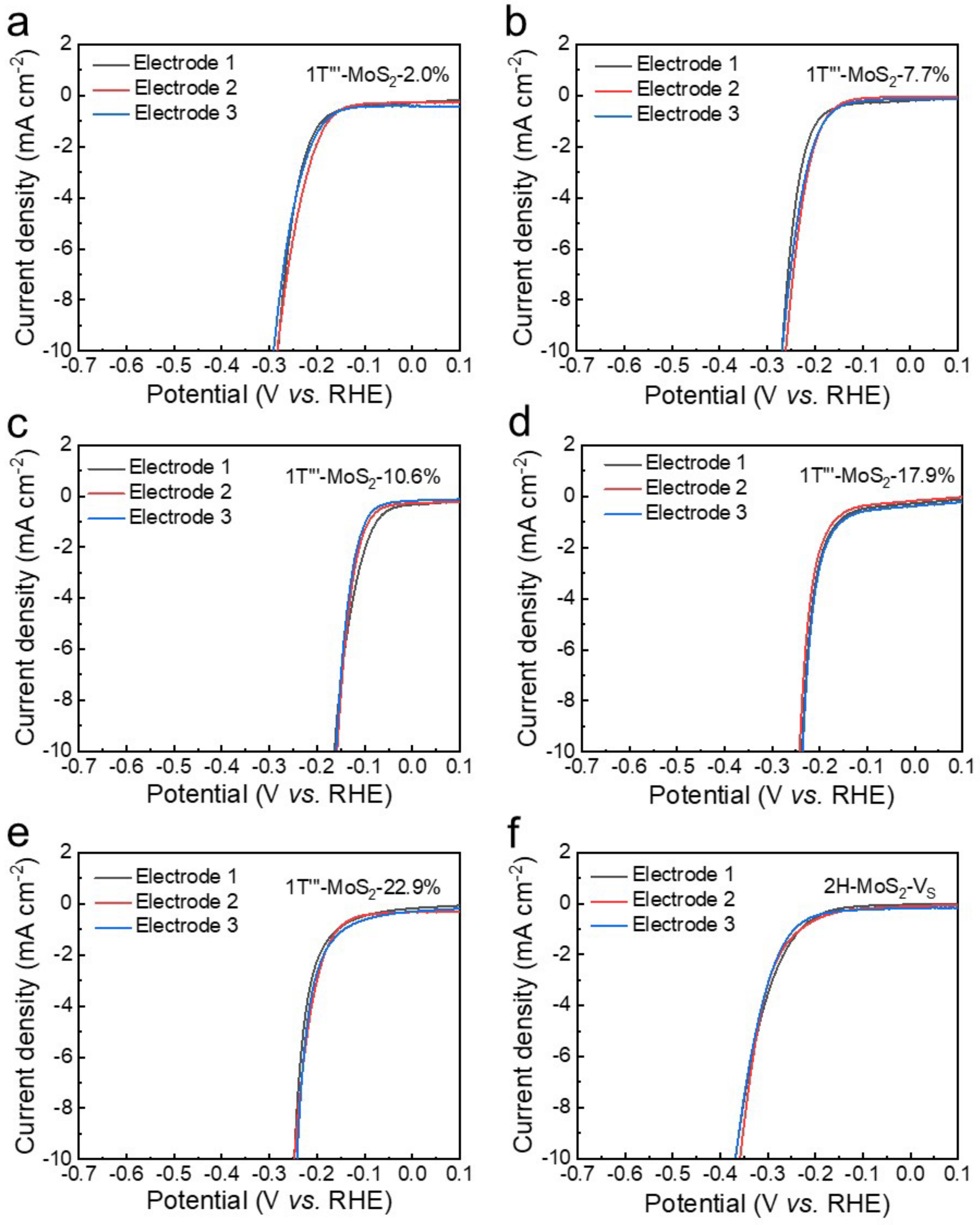

Supplementary Figure 25. HER performance tests of three electrodes per experiment. LSV curves of (a) 1T'''-MoS_2-2.0%, (b) 1T'''-MoS_2-7.7%, (c) 1T'''-MoS_2-10.6%, (d) 1T'''-MoS_2-17.9%, (e) 1T'''-MoS_2-22.9% and (f) 2H-MoS_2-V_S.

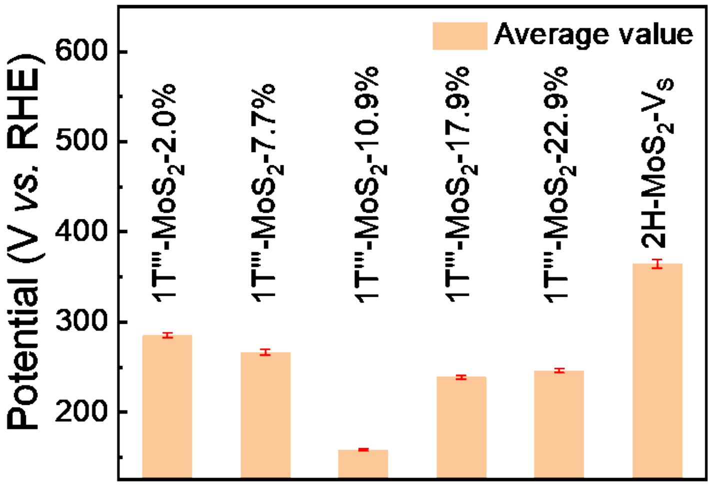

Supplementary Figure 26. Average value of overpotentials and error bars (Corresponding standard deviations). These samples correspond to 1T'''-MoS_2-V_S (V_S = 2.0%, 7.7%, 10.6%, 17.9% and 22.9%) and 2H-MoS_2-V_S (three electrodes per experiment).

Supplementary Tables

Supplementary Table 1. Crystallographic data for 1T'''-MoS_2.

Atom	x	y	z	Wyckoff.	Occ.
Mo01	0.3819 (6)	0.000000	0.5030 (15)	3*c*	1
S002	0.666667	0.333333	0.233 (3)	2*b*	1
S003	0.000000	0.000000	0.297 (4)	1a	1
S004	0.6832 (18)	0.000000	0.7661 (11)	3*c*	1

Supplementary Table 2. Crystal Data and Structural Refinement statistics for 1T'''-MoS_2.

**formula**	**MoS_2**
F_w (g mol^{-1})	160.06
crystal system	Trigonal
space group	*P*31m
a (Å)	5.580(2)
c (Å)	5.957(2)
α (°)	90
β (°)	90
γ (°)	120
V (Å^3)	160.63 (9)
Z	1
ρ_c (g·cm^{-3})	4.9612
μ (mm^{-1})	7.59
λ (Mo *K*α) (Å)	0.71073
T (K)	298
F(000)	424
θ_{max} (°) / completeness (%)	25.0 / 97.4
R_{int}	0.038
R_1[a][$F^2 > 2\sigma(F^2)$]	0.057
wR_2[b](F^2)	0.162
goodness of fit	1.22
largest diff. peak and hole (e/Å^3)	2.26 and -1.30

[a]$R_1 = \sum||F_o| - |F_c||/\sum|F_o|$. [b]$wR_2=\{\sum[w(F_o^2 - F_c^2)^2]/\sum[w(F_o^2)^2]\}^{1/2}$, $w = 1/[\sigma^2(F_o^2) + (0.1000P)^2]$ where $P = (F_o^2 + 2F_c^2)/3$.

Supplementary Table 3. Selected bond distances (Å) for 1T'''-MoS_2.[1]

Bond distance (Å)		Bond distance (Å)	
Mo—S3	2.298 (11)	Mo—S3^{iii}	2.521 (8)
Mo—S1	2.371 (14)	Mo—Mo^{iii}	3.015 (2)
Mo—S1^{i}	2.371 (14)	Mo—Mo^{ii}	3.015 (2)
Mo—S2	2.459 (12)	Mo—Mo^{iv}	3.015 (2)
Mo—S3^{ii}	2.521 (8)	Mo—Mo^{v}	3.015 (2)

Supplementary Table 4. The S/Mo atomic ratio of the 1T'''-MoS_2-V_S with different S vacancy concentrations and 2H-MoS_2-V_S.

samples	etching time (h)	S/Mo ratios	S vacancy concentrations(%)
1T'''-MoS_2-2.0%	0	1.96	2.0
1T'''-MoS_2-7.7%	0.5	1.85	7.7
1T'''-MoS_2-10.6%	1	1.79	10.6
1T'''-MoS_2-17.9%	2	1.64	17.9
1T'''-MoS_2-22.9%	3	1.54	22.9
2H-MoS_2-V_S	/	1.77	11.3

Supplementary Table 5. HER performances of as-prepared 1T'''-MoS_2-10.6% and other reported MoS_2 catalysts in acidic electrolytes.

Catalysts	Overpotential(mV) at 10mA cm^{-2}	Tafel slope ($mV\ dec^{-1}$)
1T'''-MoS_2-10.6%	158	74
SV-MoS_2[2]	170	60
Se-MoS_2 [3]	104	59
1T'-MoS_2 flakes [4]	300	83
T-MoS_2 [5]	290	78
2H c-MoS_2 [6]	191	64
S-MoS_2@C [7]	136	78
1T-MoS_2 nanosheet [8]	230	45
Defect-rich MoS_2 nanosheets [9]	192	50
50ALD (Act.)-MoS_2 [10]	348	94
mPF-MoS_2 [11]	210	74
$MoS_{2.7}$@NPG [12]	216	60
MoS_2-GNR [13]	205	50
1T-MoS_2 [14]	271	61
MoS_2 nanodots[15]	173	53

Supplementary Table 6. Average value of overpotentials measured by three electrodes at current density of 10 mA cm^{-2} of 1T'''-MoS_2-V_S (V_S = 2.0%, 7.7%, 10.6%, 17.9% and 22.9%) and 2H-MoS_2-V_S catalysts.

	**Electrode 1 (mV)**	**Electrode 2 (mV)**	**Electrode 3 (mV)**	**Average values(mV)**	**Standard deviation(mV)**
1T'''-MoS_2-2.0%	289	284	283	285.33	2.62
1T'''-MoS_2-7.7%	269	268	262	266.33	3.09
1T'''-MoS_2-10.6%	160	157	158	158.33	1.25
1T'''-MoS_2-17.9%	238	242	237	239.00	2.16
1T'''-MoS_2-22.9%	248	247	243	246.00	2.16
2H-MoS_2-V_S	369	367	358	364.67	4.78

Supplementary References

1. Fang, Y. *et al*. Structural Determination and Nonlinear Optical Properties of New 1T'''-Type MoS_2 Compound. *J. Am. Chem. Soc.* **141**, 790-793 (2019).
2. Li, H. *et al*. Activating and optimizing MoS_2 basal planes for hydrogen evolution through the formation of strained sulphur vacancies. *Nat. Mat*. **15**, 48-53 (2016).
3. Hu, J. *et al*. Engineering stepped edge surface structures of MoS_2 sheet stacks to accelerate the hydrogen evolution reaction. *Energy Environ. Sci.* **10**, 593-603 (2017).
4. Zhang, J. *et al*. Unveiling Active Sites for the Hydrogen Evolution Reaction on Monolayer MoS_2. *Adv. Mater.* **29**, 1701955 (2017).
5. Liu, Y. *et al*. Self-optimizing, highly surface-active layered metal dichalcogenide catalysts for hydrogen evolution. *Nat. Energy*. **2**, 17127 (2017).
6. Chen, Y. *et al*. Structurally Deformed MoS_2 for Electrochemically Stable, Thermally Resistant, and Highly Efficient Hydrogen Evolution Reaction. *Adv. Mater.* **29***,* 1703863 (2017).
7. Xu, Q. *et al*. Electrocatalysts: Unsaturated Sulfur Edge Engineering of Strongly Coupled MoS_2 Nanosheet-Carbon Macroporous Hybrid Catalyst for Enhanced Hydrogen Generation. *Adv. Energy Mater*. **9**, 1802553 (2019).
8. Attanayake, N. H. *et al*. Effect of Intercalated Metals on the Electrocatalytic Activity of 1T-MoS_2 for the Hydrogen Evolution Reaction. *ACS Energy. Lett*. **3**, 7-13 (2018).
9. Xie, J. *et al*. Defect-Rich MoS_2 Ultrathin Nanosheets with Additional Active Edge Sites for Enhanced Electrocatalytic Hydrogen Evolution. *Adv. Mater.* **25**, 5807-5813 (2013).
10. Kim, Y. *et al*. In Situ Electrochemical Activation of Atomic Layer Deposition Coated MoS_2 Basal Planes for Efficient Hydrogen Evolution Reaction. *Adv. Func. Mater.* **27**, 1701825 (2017).

11. Deng, J. *et al*. Multiscale structural and electronic control of molybdenum disulfide foam for highly efficient hydrogen production. *Nat. Commun.* **8**, 14430 (2017).

12. Ge, X. *et al*. Nanoporous metal enhanced catalytic activities of amorphous molybdenum sulfide for high-efficiency hydrogen production. *Adv. Mater.* **26**, 3100-3104 (2014).

13. Ekspong, J. *et al*. Stable Sulfur-Intercalated 1T' MoS_2 on Graphitic Nanoribbons as Hydrogen Evolution Electrocatalyst. *Adv. Func. Mater.* **28**, 1802744 (2018).

14. enson, E. E. *et al*. Balancing the Hydrogen Evolution Reaction, Surface Energetics, and Stability of Metallic MoS_2 Nanosheets via Covalent Functionalization. *J. Am. Chem. Soc.* **140**, 441-450 (2018).

15. Tan, C. *et al*. Preparation of high-percentage 1T-phase transition metal dichalcogenide nanodots for electrochemical hydrogen evolution. *Adv. Mater.* **30**, 1705509 (2018).
